# The redox-sensitive transcription factor Rap2.4a controls nuclear expression of 2-Cys peroxiredoxin A and other chloroplast antioxidant enzymes

**DOI:** 10.1186/1471-2229-8-48

**Published:** 2008-04-26

**Authors:** Jehad Shaikhali, Isabelle Heiber, Thorsten Seidel, Elke Ströher, Heiko Hiltscher, Stefan Birkmann, Karl-Josef Dietz, Margarete Baier

**Affiliations:** 1 Plant Biochemistry and Physiology, Bielefeld University, 33501 Bielefeld, Germany; 2 Plant Science, Heinrich-Heine-University Düsseldorf, 40225 Düsseldorf, Germany

## Abstract

**Background:**

The regulation of the chloroplast antioxidant capacity depends on nuclear gene expression. For the 2-Cys peroxiredoxin-A gene (2CPA) a *cis*-regulatory element was recently characterized, which responds to photosynthetic redox signals.

**Results:**

In a yeast-one-hybrid screen for *cis*-regulatory binding proteins, the transcription factor Rap2.4a was isolated. Rap2.4a controls the transcript abundance of the prominent chloroplast antioxidant enzyme through binding to the CGCG core of a CE3-like element. Rap2.4a activity is regulated by dithiol/disulfide transition of regulatory cysteinyl residues and subsequent changes in the quaternary structure. The mid-point redox potential of Rap2.4a activation is -269 mV (pH 7.0).

**Conclusion:**

The redox sensitivity of Rap2.4a establishes an efficient switch mechanism for redox control of nuclear gene activity of chloroplast antioxidants, in which Rap2.4 is a redox-sensor and a transducer of redox information.

## Background

In photosynthesis, excess excitation energy supports formation of reactive oxygen species (ROS) [1] which can damage metabolites, enzymes and structures [2]. Antioxidant enzymes detoxify ROS, dissipate excess energy and regenerate the electron acceptors NADP^+ ^and thioredoxin. As part of the acclimation to unfavourable growth conditions, expression of antioxidant enzymes increases under moderate stress conditions [3]. Under severe stress conditions gene expression decreases [4]. In addition, various antioxidant enzymes, such as ascorbate peroxidases (APx) [5] and peroxiredoxins [6], are inactivated. Accumulating ROS decrease the photosynthetic activity [1] and activate cytosolic defence mechanisms [1,7,8].

In CuZn-superoxide dismutase (Csd) knock-down lines of Arabidopsis, photooxidative stress alters strongest the expression pattern of chloroplast proteins [9]. Consistently, in 2-Cys peroxiredoxin (2-CP) antisense lines the imbalance in the chloroplast redox poise induces expression of chloroplast APx and monodehydroascorbate reductase [10]. *In planta *analysis of 2CPA promoter regulation [11] demonstrated that nuclear transcription of chloroplast antioxidant enzymes responds to chloroplast signals. The redox state of the plastoquinone pool [12], the redox state of low molecular weight antioxidants [13], the acceptor availability at photosystem I [4,11] and ROS [7] have been postulated to signal the chloroplast redox poise. Signal transduction through ROS, oxylipins, protochlorophyllides, metabolic coupling by carbohydrates and the redox poise of NAD(P)^+^/NAD(P)H, MAPK cascades and ABA have been indicated [14-16]. Presently, signal transduction is under intensive investigation [16,17]. First results demonstrate the signalling function of Mg^2+^-protoporphyrins and the ABA-triggered transcription factor ABI4 in correlation of nuclear gene expression with chloroplast development upon greening [14-16]. However, the precise molecular mechanisms regulating nuclear expression of chloroplast antioxidant enzymes in green tissues in a redox-dependent manner are still elusive.

Mutants screened for low expression of the nuclear encoded chloroplast 2CPA (*rimb-mutants*) differentiated transcriptional regulation of chloroplast antioxidant enzymes from typical responses to ROS accumulation, such as the induction of lipoxygenase-2 (Lox2), ascorbate peroxidase-2 (Apx2), BAP1 and Fer1 [18] [Heiber et al., unpublished data]. Many genes for cytosolic antioxidant enzymes, such as Apx2, are gradually induced to very high levels. The expression intensity correlates with the availability of the regulating transcription factor [8]. In contrast, expression of most chloroplast antioxidant enzymes is induced up to a certain stress level, but decreased in response to severe oxidative stress conditions, such as application of high concentrations of H_2_O_2 _[4,9,19] [Heiber et al., unpublished data], it is hypothesized that either a plus-minus regulator or interacting antagonistic signalling pathways control gene expression.

In respect of transcriptional regulation, the 2CPA is one of the best studied genes encoding a chloroplast antioxidant enzyme. Transcription is strongest in young developing tissues [20]. On top of the developmental regulation, the transcription intensity correlates with the acceptor availability at photosystem-I (PS-I) [11], which defines the reduction states of NADP^+ ^and thioredoxins [21]. *In planta *promoter analysis demonstrated that photosynthetic redox signal is sensed in a distinct promoter region. The target motif is located upstream of the 314 bp core promoter, that correlates 2CPA expression with chloroplast development [11]. Various nuclear encoded chloroplast proteins are co-regulated with 2CPA [18]. Piippo et al. [4] postulated that the reducing site of PS-I actually is a major signal initiation point in chloroplast-to-nucleus signaling.

A 216 bp redox-sensitive *cis*-regulatory region has been identified in the 2CPA promoter. It responds to the chloroplast redox signals [11]. Since sequence analysis gave no indication for interaction with a known redox-regulated transcription factor, a yeast-one-hybrid screen was performed to identify cis-regulatory proteins. Here isolation and characterization of the transcription factor Rap2.4a is presented. Rap2.4a is redox-sensitive, binds to a CE3-like element in the redox-sensitive promoter region and regulates transcription of 2CPA. Analysis of Rap2.4a-KO lines demonstrated that the transcription factor also impacts on expression of other nuclear encoded chloroplast antioxidant enzymes and protects plants against mild stresses, such as fluctuating environmental light conditions.

## Results

### Isolation of Rap2.4a

To identify cis-regulatory proteins involved in transcriptional regulation of 2CPA, a yeast-one-hybrid (Y1H) screen was performed with the 216 bp redox-active 2CPA promoter domain [11] and its flanking regions. The bait element was cloned into the vector pONE-1 upstream of the Gal1,10 minimal promoter and the HIS3-cDNA. The vector was transformed into the yeast strain Y187. Preys were provided by co-transformation of Y187 with a cDNA library derived from an *Arabidopsis thaliana *cell suspension culture [22]. Strongest interaction with the 2CPA promoter DNA was observed with pAct2-clone1, which encodes a fusion protein of the Gal4-activation domain (AD) and the AP2-type transcription factor Rap2.4a (At1g36060; type Ib-ERF) [23] spaced by 11 amino acids (AD-Rap2.4a).

The interaction of the AD-Rap2.4a-fusion protein with the bait was confirmed on media supplemented with 40 mM 3-amino-1,2,4-triazol (3AT), which is an inhibitor of His-biosynthesis. To exclude epigenetic regulation, the yeast strain Y187 was retransformed with *E. coli*-amplified prey and bait constructs. Survival on 40 mM 3AT confirmed the strong interaction between the transcription factor and the target element.

### Localization of the Rap2.4a-binding site in the 2CPA promoter

**Figure 1:**
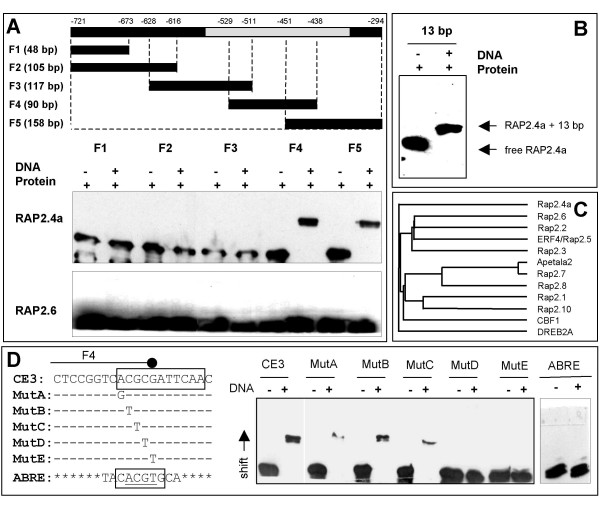
***In vitro *characterisation of DNA binding of recombinant Rap2.4a to the redox box of the 2CPA promoter**. **(A) **The 2CPA promoter region used in the Y1H-screen was amplified into 5 fragments by PCR (F1 – F5). Electrophoretic mobility shift assay (EMSA) was performed with 2.5 μg heterologously expressed His-tagged Rap2.4a or Rap2.6. The proteins were detected with anti-His antibody on Western-blots. **(B) **EMSA with a synthetic double-stranded oligonucleotide corresponding to 13 bp overlap of the fragments F4 and F5 **(C) **Similarity between AP-domain of Rap2.4a and other AP2-transcription factors according to PHYLIP. The maximal sequence variation is 22 %. **(D) **EMSA with the wild-type CE3-like element, its mutagenised variants MutA – MutE and an ABRE with His-tagged Rap2.4a followed by immunodetection with anti-His-antibody.

With five overlapping DNA-fragments (F1, F2, F3, F4 and F5) covering the Y1H-bait, the binding site of Rap2.4a was mapped to the 13 bp overlap of F4 and F5 by EMSA under non-reducing conditions (Fig. 1A). The Rap2.4a target sequence was confirmed with a synthetic double-stranded 13 bp oligonucleotide (Fig. 1B). Heterologously expressed Rap2.6 (At1g43160) (Fig. 1A), which shares 80 % sequence identity with Rap2.4a in the DNA-binding site (Fig. 1C), and control lysates of *E. coli*, which were transformed with an empty expression vector (data not shown), did not shift any 2CPA promoter fragment. Alternative to monitoring the gel shifts by immunodetection of His-tagged proteins, the interaction between the bait element and Rap2.4a was analysed by detection of biotinylated PCR-products using horseradish peroxidise-coupled streptavidin (data not shown). Here, immunodetection of the proteins was chosen as routine method, since both methods showed the interaction of DNA and proteins, but immunodetection of His-tags turned out to be easier and more efficient to apply.

Pattern analysis by MatInspector [24] predicted a coupling element 3 (CE3)-like motif (CACGCGATTC) in the 13 bp target sequence. The motif deviates in the two bases following the CGCG-core from the typical CE3-element [25] (ACGCGTGTC). Replacing the CGCG-core by TTGT abolished binding of Rap2.4a to double-stranded 20 bp oligonucleotides (data not shown), like single nucleotide substitutions of C_3 _and G_4 _did (Fig. 1D). It is concluded that C_3 _and G_4 _of the CGCG-core are essential for Rap2.4a binding.

CE3s often confer ABA-responsiveness along with ACGT-ABREs [25,26]. For example, TRAB1 can alternatively recognize the two motifs [25] and Rap2.4b can take over DREBP and ERF-function if overexpressed [27]. However, Rap2.4a neither bound the ACGT-variant of the CE3 (Mut D, Fig. 1D) nor the ABRE predicted 282 bp upstream of the CE3-like element (Fig. 1D) demonstrating the specificity of Rap2.4a for the CE3-like element.

### *In vivo *function of C_3_G_4 _in Rap2.4a-regulation of 2CPA transcription

**Figure 2:**
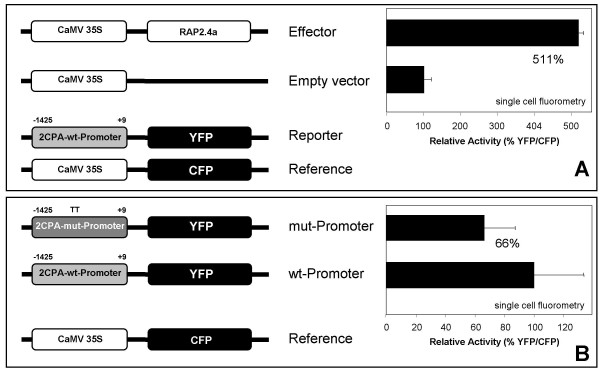
**Transactivation of the 2CPA promoter by Rap2.4a. (A) Activation of the 2CPA promoter by Rap2.4a**. Arabidopsis mesophyll protoplasts were co-transfected with plasmids encoding Rap2.4a and CFP under control of the CaMV35S-promoter and a reference plasmid encoding YFP under control of the 2CPA promoter. YFP and CFP fluorescence were quantified from 40 cells each by confocal laser scanning microscopy (CLSM) and compared to protoplasts co-transfected with a control CaMV35S plasmid and the reporter and reference constructs. (B) ***In vivo *analysis of the function of C_3_G_4_in 2CPA promoter activation by Rap2.4a**. Arabidopsis mesophyll protoplasts were co-transfected with reporter gene constructs expressing YFP either under control of the wild-type 2CPA promoter or a mutagenized 2CPA promoter, in which C_3_G_4 _was replaced by TT. The YFP fluorescence was quantified from 40 cells by CLSM and standardized on CFP activity expressed under control of the CaMV35S promoter. The averages were significantly different according to Student's t-Test (a ≤ 10%).

The regulatory function of Rap2.4a on the 2CPA promoter was tested by transient Rap2.4a over-expression (35S:Rap2.4a) in Arabidopsis mesophyll protoplasts which were transfected with 2CPA_wt_:YFP. Standardized on co-transfected 35S:CFP, 16 h Rap2.4a over-expression resulted in ca. 5-fold higher YFP activity than in an empty vector control (Fig. 2A) demonstrating that Rap2.4a is an activating transcription factor.

To test the *in vivo *function of C_3_G_4 _of the CE3-like element on Rap2.4a activation of 2CPA transcription, Arabidopsis mesophyll protoplasts were transfected either with reporter constructs expressing YFP under control of the wild-type promoter (2CPA_wt_:YFP) or a mutagenized promoter (2CPA_mut_:YFP), in which TT substituted for C_3_G_4 _(Fig. 2B). After normalization on the expression of co-transfected 35S:CFP reference constructs the YFP/CFP expression ratio of the TT-variant (2CPA_mut_:YFP) was decreased by 34 % compared to 2CPA_wt_:YFP after 16 h incubation (Fig. 2B) confirming the regulatory function of the two nucleotides in stabilization of the interaction between the transcription factor and the promoter, however it did not fully omit 2CPA promoter activation.

### Localization of Rap2.4a in Arabidopsis mesophyll protoplasts

**Figure 3:**
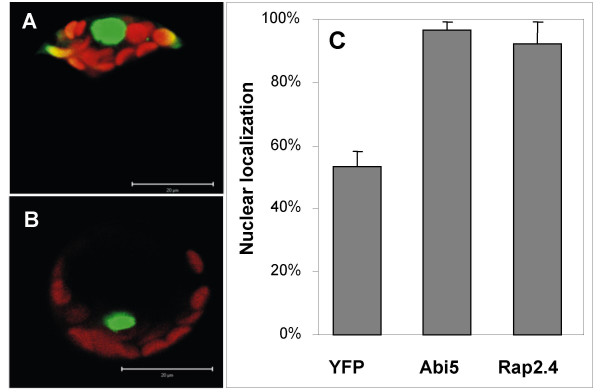
**Localization of Rap2.4a-YFP (A) and ABI5-YFP (B) in Arabidopsis mesophyll protoplasts**. green: YFP fluorescence; red: chlorophyll fluorescence. **(C) Relative YFP activity in the nucleus **as calculated from integration of the signal strength in 2D images (n = 8 - 10).

Since Rap2.4a lacks a strong nuclear localization signal and chloroplast targeting has been suggested for the Ib-ERF Rap2.4c (At2g22200) [28], the distribution of Rap2.4a protein was analysed in Arabidopsis protoplasts expressing Rap2.4a-YFP fusion proteins by confocal laser scanning microscopy (CLSM) (Fig. 3). After 12 h incubation, the majority of the protein (92 ± 7 %) was observed in the nucleus like for the YFP-fusion protein of the basic helix-loop-helix transcription factor ABI5 (97 ± 2 %), while only 53 ± 5 % of free YFP was detected in the nucleus (Fig. 3).

### Redox regulation of Rap2.4a-dependent 2CPA transcription

Luciferase reporter elements have a lower stability and higher sensitivity and time-resolution than fluorescence proteins [29]. Hence, redox regulation of the 2CPA promoter was studied in a transgenic 2CPA:luciferase line [11]. The luciferase activity was 1.8-fold increased in 35S:Rap2.4a transfected protoplasts compared to 35S:CFP-transfected ones after 2 h incubation (Tab. 1). The quick response demonstrated fast and strong activation of 2CPA by Rap2.4a.

**Table 1:** Redox regulation of Rap2.4 transcriptional activation

	Control	1 mM H_2_O_2_	1 mM DTT	1 mM Asc
35S:Rap2.4a	180 ± 30 %*	279 ± 56 %*	67 ± 11 %*	63 ± 10 %*
35S:CFP	100 ± 24 %	137 ± 18 %*	68 ± 9 %*	61 ± 11 %*
Rap2.4a/CFP	180 ± 29 %*	203 ± 38 %*	99 ± 10 %	103 ± 11 %

Relative luciferase activity (mean ± SD) in mesophyll protoplasts from transgenic Arabidopsis expressing luciferase under control of the 2CPA promoter, 2 h after transfection with either 35S:Rap2.4a or 35S:CFP, and 1.5 h after application of 1 mM H_2_O_2_, DTT and ascorbate, respectively. Significance of difference (P > 0.01; Student's T-Test; n = 72) is indicated by asterisks.

To test the function of Rap2.4a in redox-regulation of 2CPA expression, the cellular redox poise was decreased by application of 1 mM DTT or ascorbate. The antioxidants decreased the luciferase activity in Rap2.4a over-expressing protoplasts by 63 % and 65 %, respectively, within 90 min (Tab. 1). 1 mM H_2_O_2 _increased the luciferase activity in 35S:Rap2.4a lines by 54 % compared to control conditions (Tab. 1). With H_2_O_2_-concentrations higher than 3 mM the reporter gene activity decreased again indication inactivation (data not shown). Under control conditions and in 1 mM H_2_O_2_-treated samples, transfection with Rap2.4a cDNA resulted in 180 % and 203 % of the luciferase activity observed in the CFP transfected protoplasts. It is concluded that Rap2.4a activates the reporter gene. In contrast, the reporter gene activity was decreased in DTT and ascorbate treated samples. This observation suggested that Rap2.4a binding is redox-dependent.

Redox regulation of the Rap2.4a DNA binding was studied with 2 μg Rap2.4a and 100 pmol F5 in presence of either 5 mM DTT or H_2_O_2 _relative to an untreated control. DNA-binding was analysed fluorometrically by quantitative PCR after separation on agarose gels. In H_2_O_2_-treated samples 310 ± 90 % of F5 was detected compared to untreated controls. In DTT-treated samples, the amount of free F5 was 730 ± 140 % that of untreated samples (Tab. 2), demonstrating that both, H_2_O_2 _and DTT abolished DNA binding.

**Table 2:** Redox regulation of the DNA-binding affinity of Rap2.4

Control	5 mM H_2_O_2_	5 mM DTT
100 ± 18 %	309 ± 87 % *	728 ± 144 % *

The relative amount of unbound DNA (mean ± SD) after incubation of 2 μg Rap2.4a with 100 pmol F5 in 0.5 × TBE of 0.5 × TBE supplemented with 5 mM DTT or H_2_O_2_, gel extraction of unbound F5, PCR amplification and fluorometric detection on agarose gels. Significance of difference is indicated by asterisks (P > 0.05; Student's T-Test; n = 5)

### Redox regulation of Rap2.4a protein

**Figure 4:**
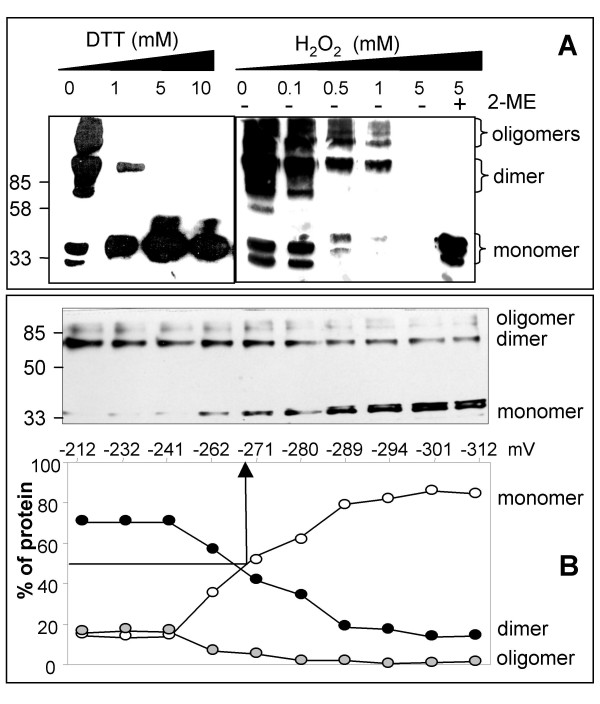
**Quaternary structure regulation of Rap2.4a under oxidizing and reducing conditions**. **(A) **RAP2.4a was incubated in the presence of DTT and H_2_O_2 _before separation on 12% SDS-PAGE and Western blot analysis using anti-HIS antibody. Rap2.4a oligomers monomerized with increasing DTT concentration. Oxidation by H_2_O_2 _resulted in oligomeric complexes with low transfer efficiency. From a series with gradually increasing DTT and H_2_O_2 _concentrations, the key steps are presented. Monomeric Rap2.4a was detected after reduction with β-mercaptoethanol. **(B) **Redox titration of Rap2.4a. The quaternary structure of heterologously expressed His-tagged Rap2.4a was analysed relative to the redox poise of the medium by SDS-PAGE separation, Western blotting and detection with anti-His antibody. The band intensities were quantified using the GelScan software package (BIOSCITECH, Marburg, Germany). The redox potential of Rap2.4a was determined from the value at which the complex was 50% dissociated.

On SDS-PAGE recombinant Rap2.4a separated with apparent molecular masses of 36 kDa, 70 kDa and larger aggregates indicating monomeric, dimeric and oligomeric forms of the transcription factor (Fig. 4). DTT abolished the oligomers, reduced the dimeric fraction and increased the amount of monomer (Fig. 4 left). After application of mild H_2_O_2 _concentrations (Fig. 4 right) only high molecular weight bands were detected. Treatment with 5 mM H_2_O_2 _resulted in a complete loss of Rap2.4a signals on the Western-Blots indicating that either high molecular mass complexes were formed, which did not migrate into the gel any more or that large protein aggregates were formed that there were beyond the transfer limits of Western-blotting. To test whether aggregates were formed and whether they were reversible, the samples were treated with 5 mM of the reductant β-mercaptoethanol minutes after the incubation with 5 mM H_2_O_2_. With β-mercaptoethanol, high amounts of monomeric Rap2.4a were detected demonstrating that H_2_O_2 _by its own formed reversible high molecular weight complexes (Fig. 4 right).

Cysteine residues are typical targets for redox regulation of proteins. Rap2.4a contains 3 cysteine residues at the positions 113, 286 and 302, which are located outside of the DNA-binding motif (aa 141 – 209) in the N- and C-terminal domains. They are not conserved in the Ib-ERF-subfamily of AP2-family of transcription factors and specific to Rap2.4a (data not shown). By equilibrium-based redox titration the midpoint redox potential of -269 mV was determined for the transition from Rap2.4a monomers to dimers (Fig. 4B). The relative amount of oligomers started to increase at redox potentials higher than -262 mV (Fig. 4B) consistent with the observation that oligomerization occurred at oxidizing conditions (Fig. 4A). Due to the difficulties to blot the aggregates quantitatively (see Fig. 4A) and the high number of different aggregates formed (Fig. 4A), it is impossible to determine the precise redox poise for the induction of aggregation. From the observations presented in Fig. 4A and 4B, it is suggested that it is a gradual multi-step process promoted by highly oxidizing conditions.

### Effect of Rap2.4a deletion on gene expression using T-DNA insertion lines

**Figure 5:**
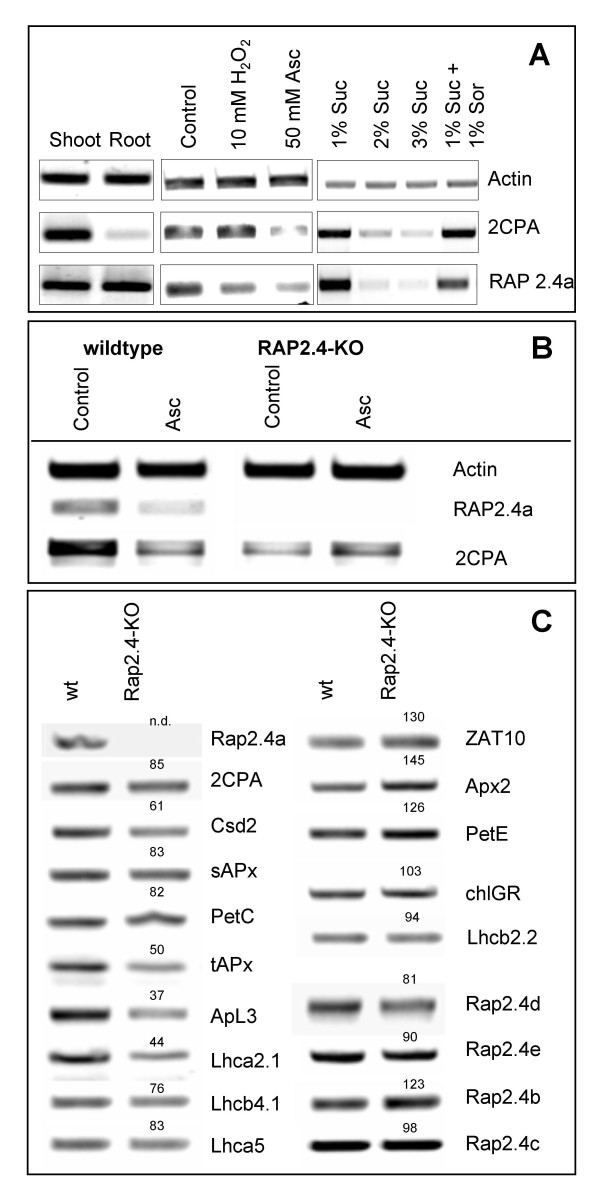
**(A) Transcript abundance of 2CPA and RAP2.4a **as analysed by RT-PCR with gene specific primers from cDNA samples standardized on actin-2 transcript amounts in leaf slices of 3 week old Arabidopsis plants in response to 4h treatment with 10 mM H_2_O_2 _and 50 mM ascorbate and in 10 day old seedlings grown on MS-medium supplemented with sucrose (Suc) and sorbitol (Sor) as indicated. **(B) RT-PCR with gene specific primers **was performed with cDNA of wild-type plants and the T-DNA-insertion line Rap2.4a-KO in samples standardized on actin-2 transcript amounts. Half of the leaf slices were treated with 50 mM ascorbate for 4 h. **(C) RT-PCR analysis of selected genes with gene specific primers **in Arabidopsis wild-type plants and Rap2.4a-KO in actin-2 normalized samples. The numbers give the transcript level relative to the control, which was set as 100.

Rap2.4a is expressed in roots and shoots (Fig. 5A left). Like the 2CPA transcript amount, Rap2.4a mRNA levels decreased in leaves upon application of 50 mM ascorbate (Fig. 5A middle) and sugars (Fig. 5A right).

The *in planta *function of Rap2.4a in 2CPA expression was assessed in T-DNA insertion lines of *Arabidopsis thaliana*. The Rap2.4a gene is interrupted upstream of the AP2-type DNA-binding site (Salk_091212; Rap2.4a-KO). Consequently, Rap2.4a-KO-lines lacked Rap2.4a mRNA (Fig. 5B). From the F2 progeny of the backcross and from the T2 progeny of the Salk-line several independent homozygous lines were selected for analysis.

In the Rap2.4a-KO-lines the 2CPA transcript level was decreased (Fig. 5B and 5C). In parallel, the transcript levels of Csd2, which encodes the major chloroplast Csd (At2g28190), stromal and thylakoid-bound ascorbate peroxidases (sAPx: At4g08390; tAPx: At1g77490), the stress inducible large subunit of the ADP-glucose-pyrophosphorylase (ApL3; At4g39210), the Rieske protein (PetC; At4g03280) and several transcripts encoding subunits of the light-harvesting complex (e.g. Lhcb4.1 (At5g01530), Lhca2.1 (At3g61470) and Lhca5 (At1g45474) were less than in wild-type plants (Fig. 5C). Although 2CPA transcription is suppressed by ascorbate in wild-type plants [11], (Fig. 5B), the transcript level was increased in Rap2.4a-KO lines (Fig. 5B).

The transcript levels of selected genes known to be induced by ROS, e.g. the transcription factor ZAT10 (At1g27730) and the cytosolic ascorbate peroxidase Apx2 (At3g09640) were slightly increased in Rap2.4a-KO, like the transcript levels of plastocyanin (PetE: At1g20340) and the ethylene-inducible DREBP-analogous Rap2.4b (At1g78080; Lin et al., 2007). The transcript amount of three other Ib-ERF transcription factors including the chloroplast targeted Ib-ERF Rap2.4c (At2g22200) [28], and that of chloroplast GR were only by 2 – 19 % decreased compared to wild-type plants.

### Effect of Rap2.4a disruption on environmental stability

**Figure 6:**
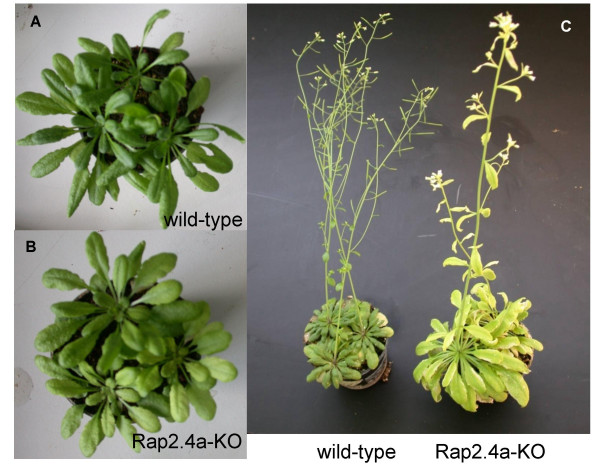
**Phenotype of Rap2.4a-KO and wild-type plants **after 8 weeks growth under controlled short-day conditions (**A**: wild-type; **B: **Rap2.4-KO) and **(C) **growth under naturally fluctuating light conditions after 3 days at maximum light intensities of 80 μmol quanta m^-2 ^s^-1 ^followed by 3 sunny days with maximum light intensities of 500 μmol quanta m^-2 ^s^-1^.

After adaptation to controlled environmental conditions, the Rap2.4a-KO T-DNA-insertion lines showed only subtile phenotypes: The chlorophyll levels were slightly decreased and the leaf blades were 8 % larger (Fig. 6A and 6B). Three independent sets of 20 plants were pre-cultivated for 6 weeks under controlled conditions (10 h continuously 100 μmol m^-2^s^-1^). Afterwards, they were transferred to the greenhouse and exposed to naturally fluctuating light conditions. After two changes between two cloudy (maximum light intensity: 80 μmol quanta m^-2 ^s^-1^; 14 h light) and two sunny days (maximum light intensity: 500 μmol quanta m^-2 ^s^-1^; 14 h light), Rap2.4a-KO lines gradually developed stress phenotypes, such as severe chlorosis, stunted, thicker and less branched inflorescences and increased leaf blade areas (Fig. 6C). In average the chlorophyll contents were decreased by 27 ± 15 % (n = 60) compared to wild-type plants and the leaf blade area increased to 183 ± 63 % (n = 60). In total in 47 % of the Rap2.4a-KO plants the chlorophyll content was at least decreased by 50 %. In 61 % of the plants the leaf blade area of the largest 5 leaves was at least twice the size of the largest leaves of wild-type-plants grown in parallel (data not shown).

## Discussion

2CPA transcription is redox-regulated on top of a strong developmental regulation, which correlates with chloroplast development and greening [4,11,20,30]. Redox-regulation is a fine-tuning mechanism which coordinates nuclear expression of the chloroplast protein with the actual environmental parameters [4,11,20,30]. In a one-hybrid screen for *cis*-regulatory proteins binding to the redox-sensitive promoter element of the 2CPA gene, the transcription factor Rap2.4a (At1g36060) was isolated. Rap2.4a binds to a CE3-like element in a redox-dependent manner (Fig. 1; Tab. 2) and activates 2CPA expression under control and slightly oxidizing conditions (Tab. 1). Although the CE3-like element in the 2CPA promoter differs from ABRE only in one base (ACGC vs. ACGT), Rap2.4a specifically bound to the CE3-like element.

According to the nomenclature by Nakano et al. [23], who recently grouped 122 AP2-type transcription factors into 12 clades, the isolated transcription factor Rap2.4a is one of eight class-Ib-ERF proteins with conserved AP2-domains, but highly variant N- and C-termini [23]. Two class-Ib-ERFs have so far been partially characterized: Rap2.4b (At1g78080) complements DREBP and blocks ethylene signalling if it is overexpressed in Arabidopsis [27]. Rap2.4c (At2g22200) is post-translationally targeted to chloroplasts where it may take over a specific, so far unknown function [28].

Analysis of Rap2.4a knock-out lines (Fig. 5) demonstrated that Rap2.4a is, unlike Rap2.4b, not fully complemented by homologous and analogous transcription factors. Hence, Rap2.4a has a specific function in plant gene regulation. Expression of several genes was impaired in absence of Rap2.4a (Fig. 5) and Rap2.4a-free (Rap2.4-KO) plants were more susceptible to fluctuating environmental conditions (Fig. 6).

Rap2.4a binding to DNA is redox regulated. It is omitted under strongly reducing and strongly oxidizing conditions (Tab. 2). From *in vitro *analysis it has to be expected that the protein monomerizes or oligomerize, respectively, under these conditions. In between, under control and slightly oxidizing conditions, the transcription factor is in its dimeric state (Fig. 4). Since Rap2.4a activated the gene expression under these conditions (Tab. 1, Fig. 2), it is concluded that dimeric Rap2.4a stimulates promoter acitivity.

Redox regulation of proteins is often maintained though thiol-disulfide regulation. Within the Rap2.4 family of transcription factors Rap2.4a is characterized by a distinct signature of cysteinyl residues. While one cysteinyl residue, which is conserved in other group members, is missing, the cysteine residue at position 113, 286 and 302 are specific for Rap2.4a. Redox-dependent oligomerization may indicate intermolecular disulfide formation and/or structural changes that foster aggregation in hetero- or homo-complexes and finally inactivation (Fig. 4, Tab. 2).

*In vivo *and *in vitro *gene expression analysis (Fig. 2 and 5; Tab. 1 and 2) demonstrated that Rap2.4a confers redox responsiveness to the 2CPA promoter by redox-dependent binding and activation (Fig. 4 and Tab. 1 and 2). In addition, Rap2.4a availability impacts on the expression of various other nuclear encoded chloroplast proteins involved in adaptation of plants to environmental variation. Increased transcript levels of ROS-regulated ZAT10 [8] and stress-induced Rap2.4b [27] demonstrate that Rap2.4a function antagonizes activation of secondary signalling cascades which are activated at higher stress thresholds. Because no homologous Rap2.4a binding sites could have been identified in the promoters of co-regulated genes (data not shown), it is assumed that the other antioxidant enzymes are indirectly co-regulated by a so far unknown mechanism. From comparison of Rap2.4a-KO lines with 2CPA antisense lines, cross talk by the availability of 2CPA mRNA or protein can be excluded. It is more likely that Rap2.4a triggers secondary transcription factors, which in turn activate the other nuclear genes for chloroplast antioxidant enzymes.

**Figure 7:**
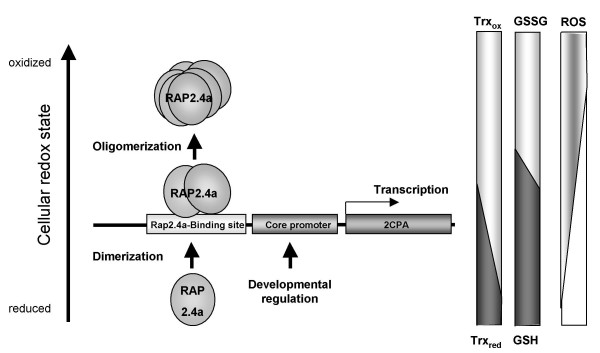
**Redox regulation of gene expression by Rap2.4a**. Nuclear expression of chloroplast antioxidant enzymes is redox-regulated by the modulation of the expression and changes in the quaternary structure of Rap2.4a. If the redox poise shifts to more oxidized values, Rap2.4a expression is increased. Rap2.4a protein dimerizes in response to the redox-shift (Fig. 4) and activates 2CPA expression. Under severe oxidative stress Rap2.4a expression is decreased and the transcription factor loses its DNA affinity and oligomerizes.

The midpoint redox potential of the activating transition from Rap2.4a monomer to dimer is -269 mV at pH 7.0 (Fig. 4B). It is more negative than the midpoint potential of glutathione (E_hc _= -230 mV), but less than that of most thioredoxins (-290 to -300 mV) [31,32]. Moderate oxidation of the glutathione pool, such as caused by photosynthetic activity and propagated by thioredoxins and the redox poise of the NAD(P)^+^/NADPH and the glutathione systems [15], may be sufficient to activate Rap2.4a-dependent gene expression. Stronger redox imbalances would inactivate Rap2.4a by aggregate formation (Fig. 4; Tab. 2) and consequently support accumulation of ROS (Fig. 5, 6, 7). While over-expression of Rap2.4a promotes 2CPA expression under control and mildly oxidizing conditions, under reducing conditions it does not impact on 2CPA expression (Tab. 1). It is concluded that Rap2.4a only confers its activating potential in its dimerized state (Fig. 7).

Rap2.4a impacts on nuclear expression of chloroplast proteins ranging from antioxidant enzymes to light-harvesting proteins (Fig. 5). The redox state of the plastoquinone pool, metabolic redox signals and ROS have been postulated to be signals indicating progressing deviation from metabolic equilibrium by increased photosynthetic electron pressure [12]. In Rap2.4-KO the transcript level of PetE, which responds to the redox state of the plastoquinone pool [12], was increased indicating a higher reduction state of the intersystem electron transport chain. Slightly increased transcript levels of the ROS-marker genes ZAT10, which encodes a strongly inducible transcription factor activating expression of cytosolic Apx [8], and its target gene Apx2, which encodes a ROS-inducible cytosolic antioxidant enzyme [33], in the Rap2.4-KO-line (Fig. 5) indicate that Rap2.4a antagonizes ROS-signalling in wild-type plants and differentiates Rap2.4a-dependent gene expression regulation from regulation of cytosolic antioxidant defence mechanisms. This observation is consistent with the analysis of the *rimb*-mutants, which were screened for decreased activation of the 2CPA promoter [18], suggesting that the regulation of genes encoding chloroplast antioxidant enzymes is independent from regulation of genes for cytosolic antioxidants. For transmission of ROS-signals, specific transcription factors, such as ZAT10 [12], have been described. Now, characterization of Rap2.4a shows the first mechanism which explains redox regulation of nuclear expression of chloroplast antioxidant enzymes.

## Conclusion

Piippo et al. [4] postulated recently that the acceptor availability at photosystem I, which regulates 2CPA transcriptional activity [11], has a stronger impact on nuclear gene expression than the redox state of the plastoquinone pool. Here, it is shown that the transcription factor Rap2.4a is a redox regulator with redox sensory function. Rap2.4a is involved in the signalling pathway triggering the 2CPA redox box [11], which responds to the acceptor availability at photosystem I [11]. The sensitivity of Rap2.4a-KO lines (Fig. 6) demonstrates that Rap2.4a regulates tolerance against natural environmental fluctuation via the control of the nuclear expression of the chloroplast antioxidant and photosynthetic systems (Fig. 5). Rap2.4a acts as a plus/minus regulator (Fig. 4 and 7, Tab. 1 and 2). Rap2.4a is activated by moderate redox imbalances, but inactivated upon severe stress (Tab. 1 and 2) consistent with decreased transcript levels for nuclear genes encoding chloroplast antioxidant after ROS-application [4,9,34]. It is concluded that Rap2.4a controls plant risk-assessment. It can switch gene expression between induction of acclimation reactions and molecular stress avoidance, such as ROS-controlled inactivation of photosynthetic electron transport.

## Methods

### Plant material and growth conditions

Plants were grown on a 1:1:1 mixture of vermiculite, pelrite and peat moss at day/night-cycles (14 h light 100 μmol quanta m^-2 ^s^-1^, 24°C and (10 h dark, 18°C) at 55% relative humidity or in the green house at natural fluctuating light conditions (14 day length; cloudy days: 20 – 80 μmol quanta m^-2 ^s^-1^; sunny days: 50 – 500 μmol quanta m^-2 ^s^-1^, 20 – 25°C). Aseptic growth on plates was performed as described in [11].

### One-hybrid screen of *Arabidopsis thaliana *cDNA library

The redox sensitive 2CPA promoter fragment [11] was amplified by PCR using the primers AAAAACTCGAGCATAATAATGAA and AAAAACTCGAGGCTTCTTCACTTA and cloned into the Xho I site of pONE1 [35] to generate the bait construct pONE1-2CPA. The yeast strain Y187 carrying pONE1-2CPA was transformed with 50 Ng of an *Arabidopsis thaliana *suspension culture cDNA library generated in pAct2 [22], selected on SD medium [36] lacking histidine, tryptophan and leucine and supplemented with 1 mM 3-aminotriazole (3AT). The clones were re-screened on SD medium containing 5 – 40 mM 3AT. Plasmids were isolated according to standard protocols [11] and transformed into *E. coli *TOP10 for further analysis.

### RNA isolation and RT-PCR

Total RNA was isolated as described in [20] and cDNA synthesis and RT-PCR performed in the linear phase of amplification according to [11] and [18] with gene specific primers, whose specificity was controlled by sequencing of the amplification products.

### Polyacrylamide gel electrophoresis (PAGE)

SDS-PAGE was performed as described in [20]. For non-reducing gels no DTT was added to the loading buffer. For native gels, the running and loading buffers were prepared without SDS and the samples were not heated prior to sample loading.

### Expression and purification of recombinant proteins

The full length cDNAs of Rap2.4a and Rap2.6 were amplified by RT-PCR using the primers Rap2.4-S (ATGGCGGATCTCTTCGGTG), Rap2.4-A (TCACGATAAAATTGAAGCCC), Rap2.6-S (ATGGTGTCTATGCTGACTAATG) and Rap2.6-A (GTTAGTTAACCAAAAGAGGAG), respectively, and cloned into pCR-T7/NT-TOPO (Invitrogen, CA). In 1 l LB cultures of transformed BL21, expression was induced at an optical density of A_600 _= 0.6-0.8 with 2 mM IPTG. After 4 h, the His-tagged proteins were affinity purified on Ni^2+^-NTA agarose resin (Qiagen, Germany).

### Electrophoretic mobility shift assays (EMSA)

2.5 μg recombinant proteins and 20 ng DNA were separated on 6–7% native polyacrylamide gels (dilution of Rotiphorese-30 (Roth, Germany) in 0.5X TBE [36] at 100 V until the bromphenolblue dye was migrated approximately 2/3 down the length of the gel. After 20 min incubation in 0.5 × TBE at room temperature it was electrophoretically blotted on nitrocellulose membranes (for protein detection) or Hybond-N^+ ^(Amersham Biosciences, UK) (for nucleic acid detection) using a semi-dry blotter. The His-tagged proteins and biotinylated PCR-products were detected with anti-His-antibody (Amersham Biosciences, UK), Light Shift Chemiluminescence EMSA Kit (Pierce, USA) and SuperSignal West Pico Chemiluminescent Substrate (Pierce, USA), respectively, according to the suppliers' protocols.

The promoter region -294 to -721 upstream of 2CPA translation start site was PCR amplified in five fragments using the primers AAACCATGGCATGCATAAGAGTC and TCCGGGAAATCCAGG for fragment 1, AAACCATGGCATGCATAAGAGTC and GATGACGGAGATGATG for fragment 2, TCTCCGTCATCGAAC and GCAGAGTTTCTGGGT for fragment 3, AAACCATGGAATACCCAGAAACT and GCGTGACCGGAGACATG for fragment 4 and CTCCGGTCACGCGATTCAAC and CTCTCTTCACTTGGTTTAC for fragment 5. To generate double-stranded 13–20 bp DNA fragments, matching synthetic single-stranded oligonucleotides were annealed at room-temperature.

### Analysis of the DNA-binding affinity of Rap2.4a

For quantification of the binding affinity, 2 Ng recombinant protein was incubated for 10 min with 100 pmol fragment F5 in 0.5 × TBE or 0.5 × TBE supplemented with 5 mM DTT or H_2_O_2 _and analysed by agarose gel electrophoresis in 0.5 × TBE for unbound F5. Free F5 was quantified in 5 parallels as described for quantification of RT-PCR products. PCR amplification (10 cycles) was performed with F5 fragments eluted from the 120 – 200 bp region of the gels using the QIAquick Gel Extraction Kit (Quiagen).

### Mutagenesis of the 2CPA promoter and transient expression analysis

Oligonucleotide-directed mutagenesis was performed according to [37] from 2CPA:LUC plasmids [11] with 2CPA-HindIII primer (GATCAATTAAGCTTAGAGTTG) and primer2-R (GTTGAAT**AA**CGTGACCGGAG) for PCR1 and primer2 (CTCCGGTCACG**TT**ATTCAAC) and 2CPA-NcoI (AGACGCCATGGCTGCTACACAC) for PCR2. The 1425 bp product was amplified from the gel purified products of PCR1 and PCR2 with 2CPA-HindIII and 2CPA-Nco I. Like the corresponding wild-type promoter (amplified with 2CPA-HindIII and 2CPA-Nco I using 2CPA:LUC [11] as template) it was cloned into the Hind III and Nco I sites of pEYFP (Clontech, CA, USA) (2CPA_wt_:YFP; 2CPA_mut_:YFP).

### Fluorometric transactivation assays and localization studies

The Rap2.4a cDNA was cloned into Age I and Eco RI sites of 35SCFP-NosT vector [38] replacing CFP. In addition, 35SCFP-NosT vector was re-ligated after Age I/Eco RI digestion to serve as empty vector control. Isolation and saturating transfection of *Arabidopsis thaliana *mesophyll protoplasts with equal amounts of 35S:Rap2.4a, 35S:CFP-NosT and 2CPA_wt_:YFP or 2CPA_mut_:YFP plasmid DNA, respectively, was performed as described in [18].

For the localization studies the cDNAs of Rap2.4a and ABI5 [39] were N-terminally fused to YFP-cDNA and expressed under the control of the CaMV35S promoter in transfected protoplasts. The distribution of YFP was alternatively quantified from 2D images taken by confocal laser scanning microscopy or using an Ascent FL fluorometer.

### Luminometric transactivation assays

The test constructs were transfected into mesophyll protoplasts isolated from the homozygous transgenic Arabidopsis line T19-2 [11] and analysed after 3 h in 100 mM sodium-phosphate buffer (pH 7.0) containing 5 mM luciferin (Duchefa) and 6 mM ATP using a Ascent FL luminometer.

### Redox titration

Redox titrations were performed with 1 μg His-tagged Rap2.4a according to [6] in 150 μl MES buffer (100 mM) supplemented with 10 mM mixtures of oxidized and reduced DTT adjusted to the respective redox potential by redox titration. At the respective equilibrium, 50 mM iodoacetamide was added, which blocks free sulfhydryl groups, to prevent thiol-disulfide exchange and inhibit post-lysis oxidation of free cysteines. The quaternary structure of Rap2.4a was visualized by non-reducing SDS-gel electrophoresis and subsequent Western blot analysis with anti-His antibody [18]. Protein amounts were quantified using the GelScan-software package (BIOSCITEC, Marburg, Germany).

### Identification and isolation of T-DNA insertion mutants

RAP2.4a (Salk_091212) T-DNA insertion lines were obtained from NASC (Loughborough, UK) and analysed for the insertion of the T-DNA in the gene of interest with the primers: Rap2.4-F (ATGGCGGATCTCTTCGGTG), Rap2.4-R1 (TCACGATAAATTGAAGCCC) and a primer for the T-DNA left border salkLBm (TGGACCGCTTGCTGCAAC).

### Accession numbers

ApL3: At4g39210; APX2: At3g09640, 2CPA: At3g11630, chlGR: At3g54660; Csd2: At2g28190; Lhca2.1: At3g61470; Lhca5: At1g45474; Lhcb2.2: At2g05070; PetC: At4g03280; PetE: At1g76100; Rap2.4a: At1g36060; Rap2.4b: At1g78080; Rap2.4c: At2g2220; Rap2.4d: At1f22190; Rap2.4e: At4g39780; sAPx: At4g08390; tAPx: At1g77490; ZAT10: At1g27730

## List of abbreviations

APx: ascorbate peroxidase; 2-CP: 2-Cys peroxiredoxin; 2CPA: (promoter of the) 2-CP A gene; aa: amino acid; ABA: abscisic acid; ABF: ABRE-binding protein; ABRE: ABA-response element; AP2: apetala-2; 3AT: 3-amino-1,2,4-triazol; CE3: coupling element 3; CFP: cyan fluorescent protein; CLSM: confocal laser scanning microscopy; Csd: CuZn superoxide dismutase; DREBP: Drought-response element binding protein; EMSA: electrophoretic mobility shift analysis; ERF: ethylene-response factor; MAPK: mitogen activate kinase; ROS: reactive oxygen species; Trx: thioredoxin; Y1H: yeast-one-hybrid; YFP: yellow fluorescent protein.

## Authors' contributions

JS carried out the yeast-one-hybrid analysis, performed the *in vitro *characterization, was involved in the CLSM analysis, in the statistical analysis and drafting of the manuscript. IH carried out the RT-PCR analysis. TS performed the CLSM experiments. ES carried out the redox titration depicted in figure 4B HH was involved in phenotyping the T-DNA insertion line. SB provided the construct for localization of Rap2.4a-GFP. KD was partially involved in designing experiments and drafting the manuscript. MB designed and coordinated the project, carried out the experiments depicted in fig. 6 and tab. 1 and 2 and drafted the manuscript.

## References

[B1] Asada K (2006). Production and scavenging of reactive oxygen species in chloroplasts and their functions. Plant Physiol.

[B2] Baier M, Dietz K-J (1999). The costs and benefits of oxygen for photosynthesizing plant cells. Progress in Botany.

[B3] Kliebenstein DJ, Monde RA, Last RL (1998). Superoxide dismutase in Arabidopsis: An eclectic enzyme family with disparate regulation and protein localization. Plant Physiol.

[B4] Piippo M, Allahverdiyeva Y, Paakkarinen V, Suoranta UM, Battchikova N, Aro EM (2006). Chloroplast-mediated regulation of nuclear genes in Arabidopsis thaliana in the absence of light stress. Physiol Genomics.

[B5] Miyake C, Asada K (1996). Inactivation mechanism of ascorbate peroxidase at low concentrations of ascorbate: Hydrogen peroxide decomposes compound I of ascorbate peroxidase. Plant Cell Physiol.

[B6] Konig J, Baier M, Horling F, Kahmann U, Harris G, Schurmann P, Dietz KJ (2002). The plant-specific function of 2-Cys peroxiredoxin-mediated detoxification of peroxides in the redox-hierarchy of photosynthetic electron flux. Proceedings of the National Academy of Sciences of the United States of America.

[B7] Rossel JB, Wilson IW, Pogson BJ (2002). Global changes in gene expression in response to high light in Arabidopsis. Plant Physiol.

[B8] Mittler R, Kim Y, Song LH, Coutu J, Coutu A, Ciftci-Yilmaz S, Lee H, Stevenson B, Zhu JK (2006). Gain- and loss-of-function mutations in Zat10 enhance the tolerance of plants to abiotic stress. Febs Letters.

[B9] Gadjev I, Vanderauwera S, Gechev TS, Laloi C, Minkov IN, Shulaev V, Apel K, Inzé D, Mittler R, Van Breusegem F (2006). Transcriptomic footprints disclose specificity of reactive oxygen species signaling in Arabidopsis. Plant Physiol.

[B10] Baier M, Noctor G, Foyer CH, Dietz KJ (2000). Antisense suppression of 2-cysteine peroxiredoxin in Arabidopsis specifically enhances the activities and expression of enzymes associated with ascorbate metabolism but not glutathione metabolism. Plant Physiol.

[B11] Baier M, Ströher E, Dietz KJ (2004). The acceptor availability at photosystem I and ABA control nuclear expression of 2-Cys peroxiredoxin-A in *Arabidopsis thaliana*. Plant Cell Physiol.

[B12] Pfannschmidt T, Allen JF, Oelmuller R (2001). Principles of redox control in photosynthesis gene expression. Physiol Plant.

[B13] Horling F, Baier M, Dietz KJ (2001). Redox-regulation of the expression of the peroxide-detoxifying chloroplast 2-Cys peroxiredoxin in the liverwort *Riccia fluitans*. Planta.

[B14] Apel K, Hirt H (2004). Reactive oxygen species: metabolism, oxidative stress, and signal transduction. Annu Rev Plant Biol.

[B15] Baier M, Dietz KJ (2005). Chloroplasts as source and target of cellular redox regulation: a discussion on chloroplast redox signals in the context of plant physiology. J Exp Bot.

[B16] Koussevitzky S, Nott A, Mockler TC, Hong F, Sachetto-Martins G, Surpin Z, Lim IJ, Mittler R, Chory J (2007). Signals from chloroplasts converge to regulate nuclear gene expression. Science.

[B17] Pesaresi P, Schneider A, Kleine T, Leister D (2007). Interorganellar communication. Curr Opin Plant Biol.

[B18] Heiber I, Ströher E, Raatz B, Busse I, Kahmann U, Bevan MW, Dietz KJ, Baier M (2007). The redox imbalanced mutants of arabidopsis differentiate signaling pathways for redox regulation of chloroplast antioxidant enzymes. Plant Physiol.

[B19] https://iii.genevestigator.ethz.ch/at/.

[B20] Baier M, Dietz KJ (1996). Primary structure and expression of plant homologues of animal and fungal thioredoxin-dependent peroxide reductases and bacterial alkyl hydroperoxide reductases. Plant Mol Biol.

[B21] Fridlyand LE, Scheibe R (1999). Controlled distribution of electrons between acceptors in chloroplasts: a theoretical consideration. Biochimica et Biophysica Acta-Bioenergetics.

[B22] Nemeth K, Salchert K, Putnoky P, Bhalerao R, Koncz-Kalman Z, Stankovic-Stangeland B, Bako L, Mathur J, Okresz L, Stabel S, Geigenberger P, Stitt M, Redei GP, Schell J, Koncz C (1998). Pleiotropic control of glucose and hormone responses by PRL1, a nuclear WD protein, in Arabidopsis. Genes Dev.

[B23] Nakano T, Suzuki K, Fujimura T, Shinshi H (2006). Genome-wide analysis of the ERF gene family in Arabidopsis and rice. Plant Physiol.

[B24] http://www.GENOMATRIX.de.

[B25] Hobo T, Asada M, Kowyama Y, Hattori T (1999). ACGT-containing abscisic acid response element (ABRE) and coupling element 3 (CE3) are functionally equivalent. Plant J.

[B26] Shen QJ, Casaretto JA, Zhang P, Ho TH (2004). Functional definition of ABA-response complexes: the promoter units necessary and sufficient for ABA induction of gene expression in barley (*Hordeum vulgare L*.). Plant Mol Biol.

[B27] Lin R-C, Rark HJ, Wanf H-Y (2007). Role of Arabidopsis RAP2.4 is regulating light- and ethylene-mediated development processes and drought stress tolerance. Mol Plant.

[B28] Schwacke R, Fischer K, Ketelsen B, Krupinska K, Krause K (2007). Comparative survey of plastid and mitochondrial targeting properties of transcription factors in Arabidopsis and rice. Molecular Genetics and Genomics.

[B29] de Ruijter NCA, Verhees J, van Leeuwen W, Krol AR van der (2003). Evaluation and comparison of the GUS, LUC and GFP reporter system for gene expression studies in plants. Plant Biology.

[B30] Pena-Ahumada A, Kahmann U, Dietz KJ, Baier M (2006). Regulation of peroxiredoxin expression versus expression of Halliwell-Asada-Cycle enzymes during early seedling development of Arabidopsis thaliana. Photosynth Res.

[B31] Aslund F, Berndt KD, Holmgren A (1997). Redox potentials of glutaredoxins and other thiol-disulfide oxidoreductases of the thioredoxin superfamily determined by direct protein-protein redox equilibria. Journal of Biological Chemistry.

[B32] Collin V, Issakidis-Bourguet E, Marchand C, Hirasawa M, Lancelin JM, Knaff DB, Dietz KJ, Issakidis-Bourguet E (2003). The Arabidopsis plastidial thioredoxins: new functions and new insights into specificity. J Biol Chem.

[B33] Karpinski S, Escobar C, Karpinska B, Creissen G, Mullineaux PM (1997). Photosynthetic electron transport regulates the expression of cytosolic ascorbate peroxidase genes in Arabidopsis excess light stress. Plant Cell.

[B34] Vandenabeele S, Vanderauwera S, Vuylsteke M, Rombauts S, Langebartels C, Seidlitz HK, Zabeau M, Van Montagu M, Inzé D, Van Breusegem F (2004). Catalase deficiency drastically affects gene expression induced by high light in Arabidopsis thaliana. Plant J.

[B35] Cormack RS, Eulgem T, Rushton PJ, Kochner P, Hahlbrock K, Somssich IE (2002). Leucine zipper-containing WRKY proteins widen the spectrum of immediate early elicitor-induced WRKY transcription factors in parsley. Biochim Biophys Acta.

[B36] Ausubel FM, Berndt KD, Holmgren A (2001). Current protocol in molecular biology.

[B37] Montemartini M, Kalisz HM, Hecht HJ, Steinert P, Flohe L (1999). Activation of active-site cysteine residues in the peroxiredoxin-type tryparedoxin peroxidase of *Crithidia fasciculata*. Eur J Biochem.

[B38] Seidel T, Kluge C, Hanitzsch M, Ross J, Sauer M, Dietz KJ, Golldack D (2004). Colocalization and FRET-analysis of subunits c and a of the vacuolar H^+^-ATPase in living plant cells. Journal of Biotechnology.

[B39] Lopez-Molina L, Mongrand S, Kinoshita N, Chua NH (2003). AFP is a novel negative regulator of ABA signaling that promotes ABI5 protein degradation. Genes Dev.

